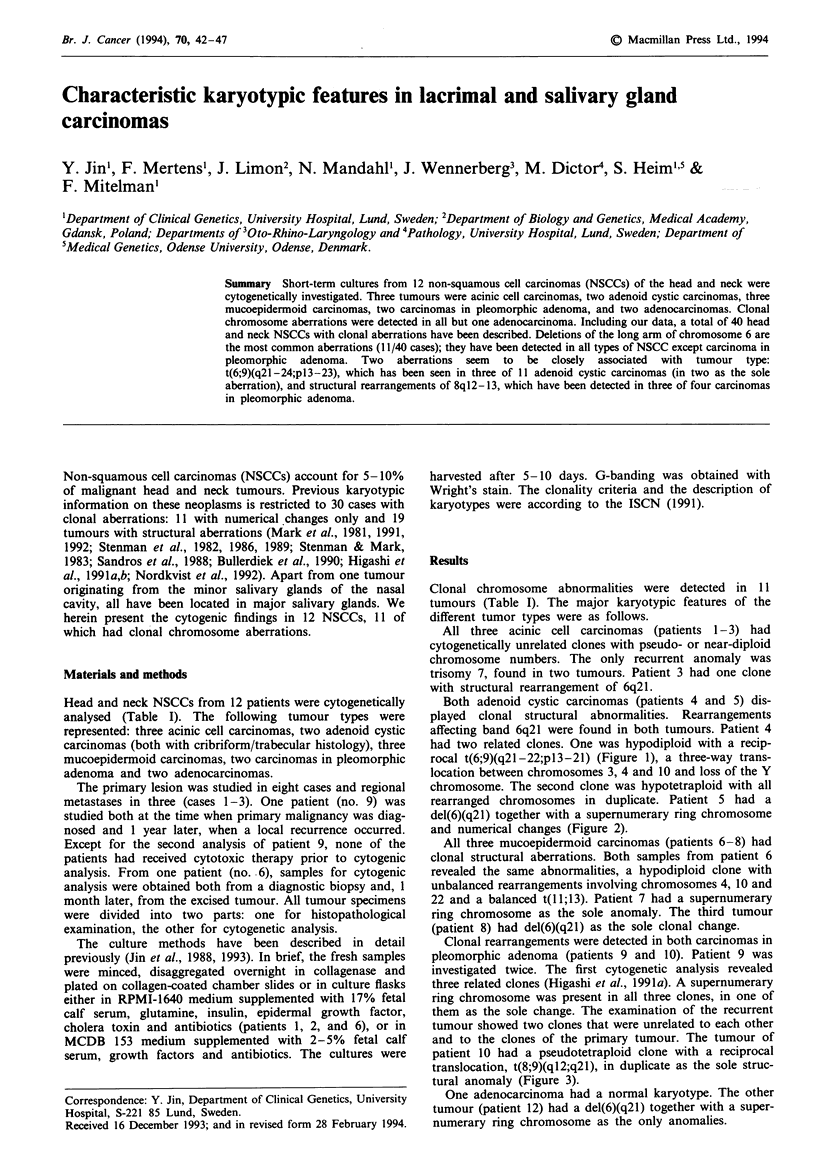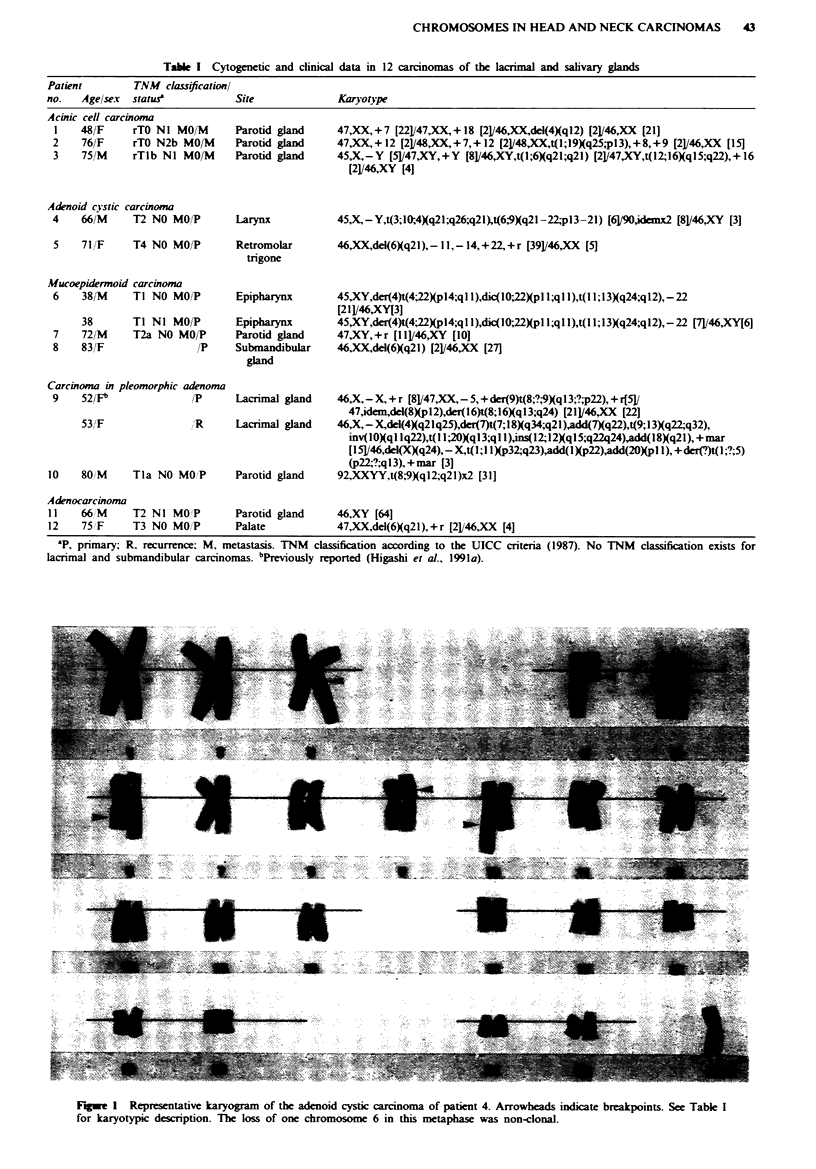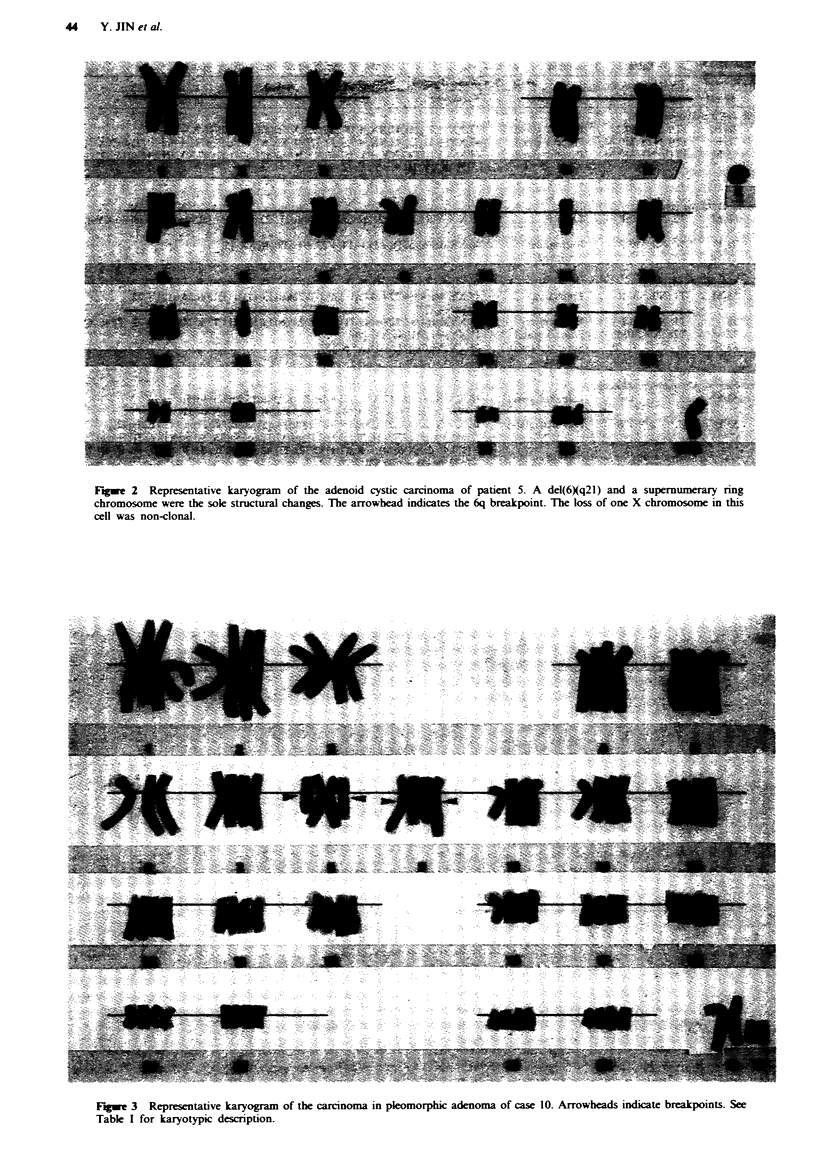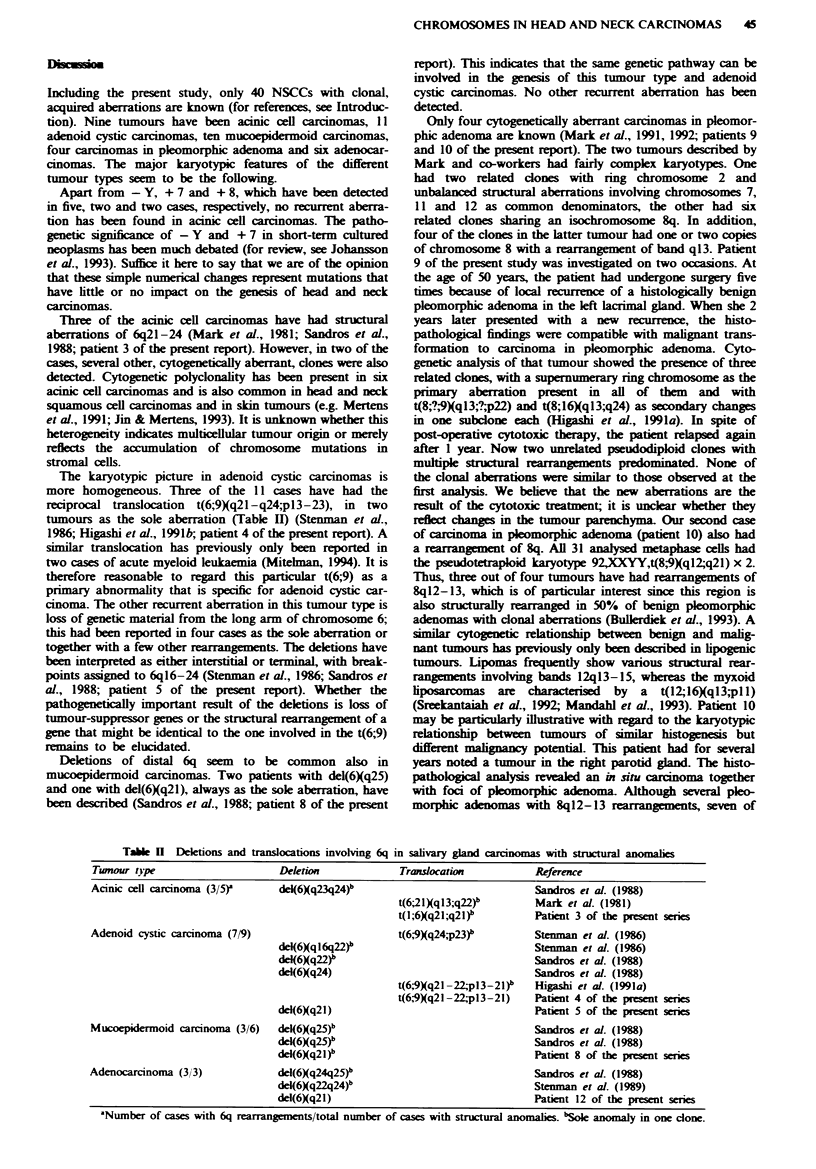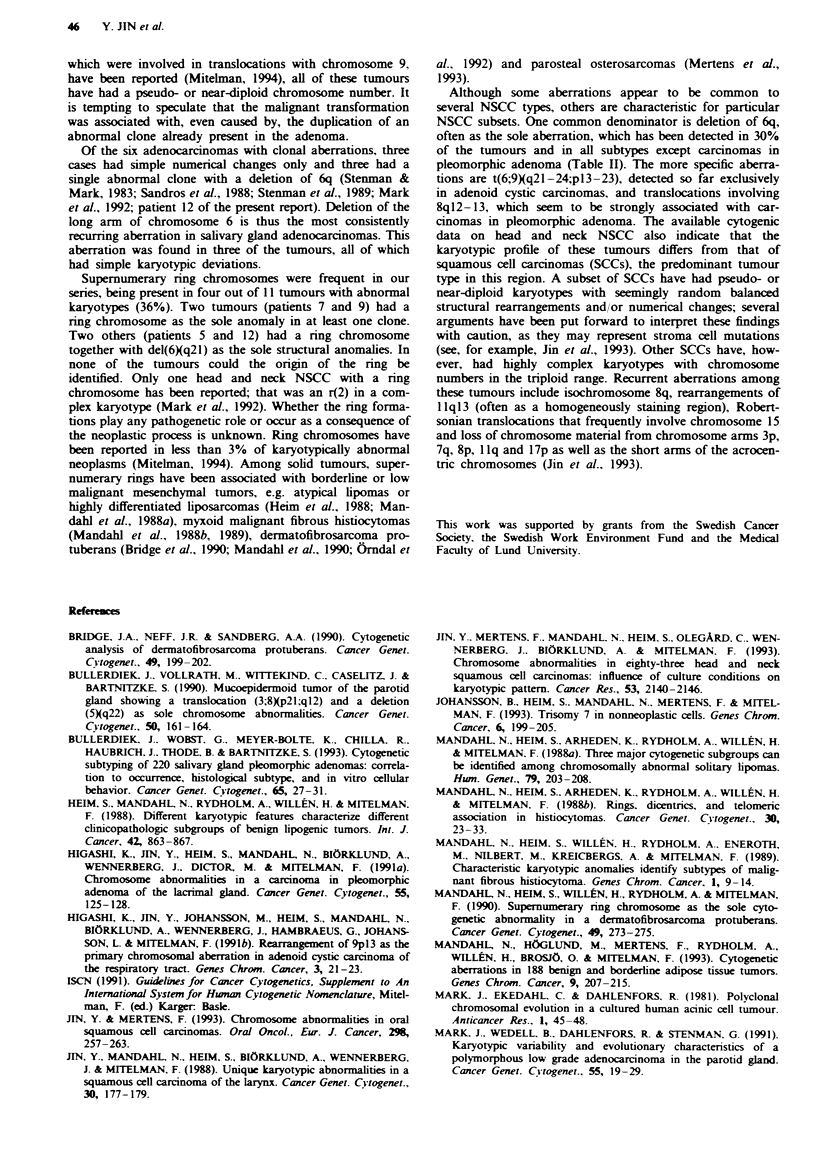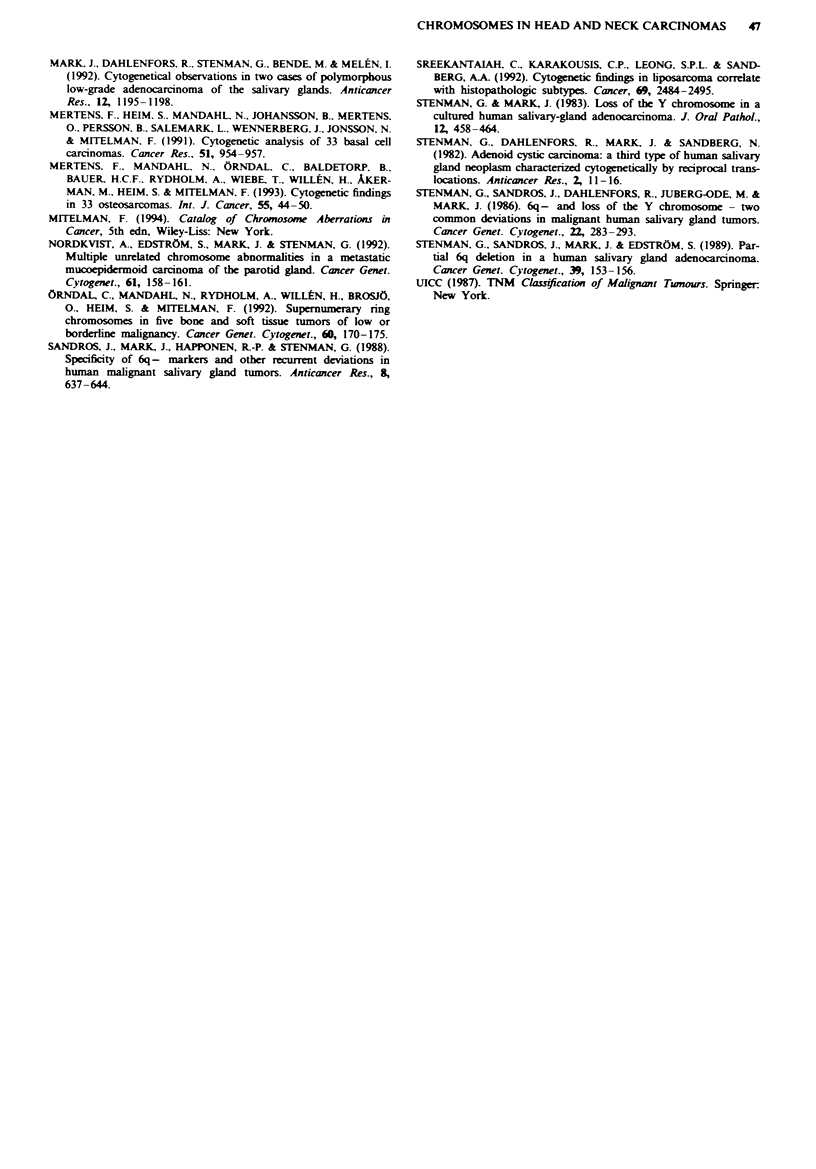# Characteristic karyotypic features in lacrimal and salivary gland carcinomas.

**DOI:** 10.1038/bjc.1994.247

**Published:** 1994-07

**Authors:** Y. Jin, F. Mertens, J. Limon, N. Mandahl, J. Wennerberg, M. Dictor, S. Heim, F. Mitelman

**Affiliations:** Department of Clinical Genetics, University Hospital, Lund, Sweden.

## Abstract

**Images:**


Br. J. Cancer (1994), 70, 42-47 © Macmillan Press Ltd., 1994

### Characteristic karyotypic features in lacrimal and salivary gland carcinomas

Y. Jin[1], F. Mertens[1], J. Limon[2], N. Mandahl[1], J. Wennerberg[3], M. Dictor[4], S. Heim[1,5] & F. Mitelman[1]

[1]*Department of Clinical Genetics, University Hospital, Lund, Sweden;* [2]*Department of Biology and Genetics, Medical Academy, Gdansk, Poland; Departments of* [3]*Oto-Rhino-Laryngology and* [4]*Pathology, University Hospital, Lund, Sweden; Department of* [5]*Medical Genetics, Odense University, Odense, Denmark.*

**Summary** Short-term cultures from 12 non-squamous cell carcinomas (NSCCs) of the head and neck were cytogenetically investigated. Three tumours were acinic cell carcinomas, two adenoid cystic carcinomas, three mucoepidermoid carcinomas, two carcinomas in pleomorphic adenoma, and two adenocarcinomas. Clonal chromosome aberrations were detected in all but one adenocarcinoma. Including our data, a total of 40 head and neck NSCCs with clonal aberrations have been described. Deletions of the long arm of chromosome 6 are the most common aberrations (11/40 cases); they have been detected in all types of NSCC except carcinoma in pleomorphic adenoma. Two aberrations seem to be closely associated with tumour type: t(6;9)(q21-24;p13-23), which has been seen in three of 11 adenoid cystic carcinomas (in two as the sole aberration), and structural rearrangements of 8q12-13, which have been detected in three of four carcinomas in pleomorphic adenoma.

Non-squamous cell carcinomas (NSCCs) account for 5-10% of malignant head and neck tumours. Previous karyotypic information on these neoplasms is restricted to 30 cases with clonal aberrations: 11 with numerical changes only and 19 tumours with structural aberrations (Mark *et al.*, 1981, 1991, 1992; Stenman *et al.*, 1982, 1986, 1989; Stenman & Mark, 1983; Sandros *et al.*, 1988; Bullerdiek *et al.*, 1990; Higashi *et al.*, 1991*a*,*b*; Nordkvist *et al.*, 1992). Apart from one tumour originating from the minor salivary glands of the nasal cavity, all have been located in major salivary glands. We herein present the cytogenic findings in 12 NSCCs, 11 of which had clonal chromosome aberrations.

### Materials and methods

Head and neck NSCCs from 12 patients were cytogenetically analysed (Table I). The following tumour types were represented: three acinic cell carcinomas, two adenoid cystic carcinomas (both with cribriform/trabecular histology), three mucoepidermoid carcinomas, two carcinomas in pleomorphic adenoma and two adenocarcinomas.

The primary lesion was studied in eight cases and regional metastases in three (cases 1-3). One patient (no. 9) was studied both at the time when primary malignancy was diagnosed and 1 year later, when a local recurrence occurred. Except for the second analysis of patient 9, none of the patients had received cytotoxic therapy prior to cytogenic analysis. From one patient (no. 6), samples for cytogenic analysis were obtained both from a diagnostic biopsy and, 1 month later, from the excised tumour. All tumour specimens were divided into two parts: one for histopathological examination, the other for cytogenetic analysis.

The culture methods have been described in detail previously (Jin *et al.*, 1988, 1993). In brief, the fresh samples were minced, disaggregated overnight in collagenase and plated on collagen-coated chamber slides or in culture flasks either in RPMI-1640 medium supplemented with 17% fetal calf serum, glutamine, insulin, epidermal growth factor, cholera toxin and antibiotics (patients 1, 2, and 6), or in MCDB 153 medium supplemented with 2-5% fetal calf serum, growth factors and antibiotics. The cultures were harvested after 5-10 days. G-banding was obtained with Wright's stain. The clonality criteria and the description of karyotypes were according to the ISCN (1991).

### Results

Clonal chromosome abnormalities were detected in 11 tumours (Table I). The major karyotypic features of the different tumor types were as follows.

All three acinic cell carcinomas (patients 1-3) had cytogenetically unrelated clones with pseudo- or near-diploid chromosome numbers. The only recurrent anomaly was trisomy 7, found in two tumours. Patient 3 had one clone with structural rearrangement of 6q21.

Both adenoid cystic carcinomas (patients 4 and 5) displayed clonal structural abnormalities. Rearrangements affecting band 6q21 were found in both tumours. Patient 4 had two related clones. One was hypodiploid with a reciprocal t(6;9)(q21-22;p13-21) (Figure 1), a three-way translocation between chromosomes 3, 4 and 10 and loss of the Y chromosome. The second clone was hypotetraploid with all rearranged chromosomes in duplicate. Patient 5 had a del(6)(q21) together with a supernumerary ring chromosome and numerical changes (Figure 2).

All three mucoepidermoid carcinomas (patients 6-8) had clonal structural aberrations. Both samples from patient 6 revealed the same abnormalities, a hypodiploid clone with unbalanced rearrangements involving chromosomes 4, 10 and 22 and a balanced t(11;13). Patient 7 had a supernumerary ring chromosome as the sole anomaly. The third tumour (patient 8) had del(6)(q21) as the sole clonal change.

Clonal rearrangements were detected in both carcinomas in pleomorphic adenoma (patients 9 and 10). Patient 9 was investigated twice. The first cytogenetic analysis revealed three related clones (Higashi *et al.*, 1991*a*). A supernumerary ring chromosome was present in all three clones, in one of them as the sole change. The examination of the recurrent tumour showed two clones that were unrelated to each other and to the clones of the primary tumour. The tumour of patient 10 had a pseudotetraploid clone with a reciprocal translocation, t(8;9)(q12;q21), in duplicate as the sole structural anomaly (Figure 3).

One adenocarcinoma had a normal karyotype. The other tumour (patient 12) had a del(6)(q21) together with a supernumerary ring chromosome as the only anomalies.

Correspondence: Y. Jin, Department of Clinical Genetics, University Hospital, S-221 85 Lund, Sweden.
Received 16 December 1993; and in revised form 28 February 1994.

**Table I** Cytogenetic and clinical data in 12 carcinomas of the lacrimal and salivary glands

| *Patient no.* | *Age/sex* | *TNM classification/ status*[a] | *Site* | *Karyotype* |
|---|---|---|---|---|
| *Acinic cell carcinoma* | | | | |
| 1 | 48/F | rT0 N1 M0/M | Parotid gland | 47,XX,+7 [22]/47,XX,+18 [2]/46,XX,del(4)(q12) [2]/46,XX [21] |
| 2 | 76/F | rT0 N2b M0/M | Parotid gland | 47,XX,+12 [2]/48,XX,+7,+12 [2]/48,XX,t(1;19)(q25;p13),+8,+9 [2]/46,XX [15] |
| 3 | 75/M | rT1b N1 M0/M | Parotid gland | 45,X,−Y [5]/47,XY,+Y [8]/46,XY,t(1;6)(q21;q21) [2]/47,XY,t(12;16)(q15;q22),+16 [2]/46,XY [4] |
| *Adenoid cystic carcinoma* | | | | |
| 4 | 66/M | T2 N0 M0/P | Larynx | 45,X,−Y,t(3;10;4)(q21;q26;q21),t(6;9)(q21−22;p13−21) [6]/90,idemx2 [8]/46,XY [3] |
| 5 | 71/F | T4 N0 M0/P | Retromolar trigone | 46,XX,del(6)(q21),−11,−14,+22,+r [39]/46,XX [5] |
| *Mucoepidermoid carcinoma* | | | | |
| 6 | 38/M | T1 N0 M0/P | Epipharynx | 45,XY,der(4)t(4;22)(p14;q11),dic(10;22)(p11;q11),t(11;13)(q24;q12),−22 [21]/46,XY[3] |
| | 38 | T1 N1 M0/P | Epipharynx | 45,XY,der(4)t(4;22)(p14;q11),dic(10;22)(p11;q11),t(11;13)(q24;q12),−22 [7]/46,XY[6] |
| 7 | 72/M | T2a N0 M0/P | Parotid gland | 47,XY,+r [11]/46,XY [10] |
| 8 | 83/F | /P | Submandibular gland | 46,XX,del(6)(q21) [2]/46,XX [27] |
| *Carcinoma in pleomorphic adenoma* | | | | |
| 9 | 52/F[b] | /P | Lacrimal gland | 46,X,−X,+r [8]/47,XX,−5,+der(9)t(8;?;9)(q13;?;p22),+r[5]/ 47,idem,del(8)(p12),der(16)t(8;16)(q13;q24) [21]/46,XX [22] |
| | 53/F | /R | Lacrimal gland | 46,X,−X,del(4)(q21q25),der(7)t(7;18)(q34;q21),add(7)(q22),t(9;13)(q22;q32), inv(10)(q11q22),t(11;20)(q13;q11),ins(12;12)(q15;q22q24),add(18)(q21),+mar [15]/46,del(X)(q24),−X,t(1;11)(p32;q23),add(1)(p22),add(20)(p11),+der(?)t(1;?;5) (p22;?;q13),+mar [3] |
| 10 | 80/M | T1a N0 M0/P | Parotid gland | 92,XXYY,t(8;9)(q12;q21)x2 [31] |
| *Adenocarcinoma* | | | | |
| 11 | 66/M | T2 N1 M0/P | Parotid gland | 46,XY [64] |
| 12 | 75/F | T3 N0 M0/P | Palate | 47,XX,del(6)(q21),+r [2]/46,XX [4] |

[a]P, primary; R, recurrence; M, metastasis. TNM classification according to the UICC criteria (1987). No TNM classification exists for lacrimal and submandibular carcinomas. [b]Previously reported (Higashi *et al.*, 1991*a*).

**Figure 1** Representative karyogram of the adenoid cystic carcinoma of patient 4. Arrowheads indicate breakpoints. See Table I for karyotypic description. The loss of one chromosome 6 in this metaphase was non-clonal.

**Figure 2** Representative karyogram of the adenoid cystic carcinoma of patient 5. A del(6)(q21) and a supernumerary ring chromosome were the sole structural changes. The arrowhead indicates the 6q breakpoint. The loss of one X chromosome in this cell was non-clonal.

**Figure 3** Representative karyogram of the carcinoma in pleomorphic adenoma of case 10. Arrowheads indicate breakpoints. See Table I for karyotypic description.

### Discussion

Including the present study, only 40 NSCCs with clonal, acquired aberrations are known (for references, see Introduction). Nine tumours have been acinic cell carcinomas, 11 adenoid cystic carcinomas, ten mucoepidermoid carcinomas, four carcinomas in pleomorphic adenoma and six adenocarcinomas. The major karyotypic features of the different tumour types seem to be the following.

Apart from – Y, + 7 and + 8, which have been detected in five, two and two cases, respectively, no recurrent aberration has been found in acinic cell carcinomas. The pathogenetic significance of – Y and + 7 in short-term cultured neoplasms has been much debated (for review, see Johansson *et al.*, 1993). Suffice it here to say that we are of the opinion that these simple numerical changes represent mutations that have little or no impact on the genesis of head and neck carcinomas.

Three of the acinic cell carcinomas have had structural aberrations of 6q21–24 (Mark *et al.*, 1981; Sandros *et al.*, 1988; patient 3 of the present report). However, in two of the cases, several other, cytogenetically aberrant, clones were also detected. Cytogenetic polyclonality has been present in six acinic cell carcinomas and is also common in head and neck squamous cell carcinomas and in skin tumours (e.g. Mertens *et al.*, 1991; Jin & Mertens, 1993). It is unknown whether this heterogeneity indicates multicellular tumour origin or merely reflects the accumulation of chromosome mutations in stromal cells.

The karyotypic picture in adenoid cystic carcinomas is more homogeneous. Three of the 11 cases have had the reciprocal translocation t(6;9)(q21–q24;p13–23), in two tumours as the sole aberration (Table II) (Stenman *et al.*, 1986; Higashi *et al.*, 1991*b*; patient 4 of the present report). A similar translocation has previously only been reported in two cases of acute myeloid leukaemia (Mitelman, 1994). It is therefore reasonable to regard this particular t(6;9) as a primary abnormality that is specific for adenoid cystic carcinoma. The other recurrent aberration in this tumour type is loss of genetic material from the long arm of chromosome 6; this had been reported in four cases as the sole aberration or together with a few other rearrangements. The deletions have been interpreted as either interstitial or terminal, with breakpoints assigned to 6q16–24 (Stenman *et al.*, 1986; Sandros *et al.*, 1988; patient 5 of the present report). Whether the pathogenetically important result of the deletions is loss of tumour-suppressor genes or the structural rearrangement of a gene that might be identical to the one involved in the t(6;9) remains to be elucidated.

Deletions of distal 6q seem to be common also in mucoepidermoid carcinomas. Two patients with del(6)(q25) and one with del(6)(q21), always as the sole aberration, have been described (Sandros *et al.*, 1988; patient 8 of the present report). This indicates that the same genetic pathway can be involved in the genesis of this tumour type and adenoid cystic carcinomas. No other recurrent aberration has been detected.

Only four cytogenetically aberrant carcinomas in pleomorphic adenoma are known (Mark *et al.*, 1991, 1992; patients 9 and 10 of the present report). The two tumours described by Mark and co-workers had fairly complex karyotypes. One had two related clones with ring chromosome 2 and unbalanced structural aberrations involving chromosomes 7, 11 and 12 as common denominators, the other had six related clones sharing an isochromosome 8q. In addition, four of the clones in the latter tumour had one or two copies of chromosome 8 with a rearrangement of band q13. Patient 9 of the present study was investigated on two occasions. At the age of 50 years, the patient had undergone surgery five times because of local recurrence of a histologically benign pleomorphic adenoma in the left lacrimal gland. When she 2 years later presented with a new recurrence, the histopathological findings were compatible with malignant transformation to carcinoma in pleomorphic adenoma. Cytogenetic analysis of that tumour showed the presence of three related clones, with a supernumerary ring chromosome as the primary aberration present in all of them and with t(8;?;9)(q13;?;p22) and t(8;16)(q13;q24) as secondary changes in one subclone each (Higashi *et al.*, 1991*a*). In spite of post-operative cytotoxic therapy, the patient relapsed again after 1 year. Now two unrelated pseudodiploid clones with multiple structural rearrangements predominated. None of the clonal aberrations were similar to those observed at the first analysis. We believe that the new aberrations are the result of the cytotoxic treatment; it is unclear whether they reflect changes in the tumour parenchyma. Our second case of carcinoma in pleomorphic adenoma (patient 10) also had a rearrangement of 8q. All 31 analysed metaphase cells had the pseudotetraploid karyotype 92,XXYY,t(8;9)(q12;q21) × 2. Thus, three out of four tumours have had rearrangements of 8q12–13, which is of particular interest since this region is also structurally rearranged in 50% of benign pleomorphic adenomas with clonal aberrations (Bullerdiek *et al.*, 1993). A similar cytogenetic relationship between benign and malignant tumours has previously only been described in lipogenic tumours. Lipomas frequently show various structural rearrangements involving bands 12q13–15, whereas the myxoid liposarcomas are characterised by a t(12;16)(q13;p11) (Sreekantaiah *et al.*, 1992; Mandahl *et al.*, 1993). Patient 10 may be particularly illustrative with regard to the karyotypic relationship between tumours of similar histogenesis but different malignancy potential. This patient had for several years noted a tumour in the right parotid gland. The histopathological analysis revealed an *in situ* carcinoma together with foci of pleomorphic adenoma. Although several pleomorphic adenomas with 8q12–13 rearrangements, seven of

**Table II** Deletions and translocations involving 6q in salivary gland carcinomas with structural anomalies

| *Tumour type* | *Deletion* | *Translocation* | *Reference* |
|---|---|---|---|
| Acinic cell carcinoma (3/5)[a] | del(6)(q23q24)[b] | | Sandros *et al.* (1988) |
| | | t(6;21)(q13;q22)[b] | Mark *et al.* (1981) |
| | | t(1;6)(q21;q21)[b] | Patient 3 of the present series |
| Adenoid cystic carcinoma (7/9) | | t(6;9)(q24;p23)[b] | Stenman *et al.* (1986) |
| | del(6)(q16q22)[b] | | Stenman *et al.* (1986) |
| | del(6)(q22)[b] | | Sandros *et al.* (1988) |
| | del(6)(q24) | | Sandros *et al.* (1988) |
| | | t(6;9)(q21–22;p13–21)[b] | Higashi *et al.* (1991*a*) |
| | | t(6;9)(q21–22;p13–21) | Patient 4 of the present series |
| | del(6)(q21) | | Patient 5 of the present series |
| Mucoepidermoid carcinoma (3/6) | del(6)(q25)[b] | | Sandros *et al.* (1988) |
| | del(6)(q25)[b] | | Sandros *et al.* (1988) |
| | del(6)(q21)[b] | | Patient 8 of the present series |
| Adenocarcinoma (3/3) | del(6)(q24q25)[b] | | Sandros *et al.* (1988) |
| | del(6)(q22q24)[b] | | Stenman *et al.* (1989) |
| | del(6)(q21) | | Patient 12 of the present series |

[a]Number of cases with 6q rearrangements/total number of cases with structural anomalies. [b]Sole anomaly in one clone.

which were involved in translocations with chromosome 9, have been reported (Mitelman, 1994), all of these tumours have had a pseudo- or near-diploid chromosome number. It is tempting to speculate that the malignant transformation was associated with, even caused by, the duplication of an abnormal clone already present in the adenoma.

Of the six adenocarcinomas with clonal aberrations, three cases had simple numerical changes only and three had a single abnormal clone with a deletion of 6q (Stenman & Mark, 1983; Sandros *et al*., 1988; Stenman *et al*., 1989; Mark *et al*., 1992; patient 12 of the present report). Deletion of the long arm of chromosome 6 is thus the most consistently recurring aberration in salivary gland adenocarcinomas. This aberration was found in three of the tumours, all of which had simple karyotypic deviations.

Supernumerary ring chromosomes were frequent in our series, being present in four out of 11 tumours with abnormal karyotypes (36%). Two tumours (patients 7 and 9) had a ring chromosome as the sole anomaly in at least one clone. Two others (patients 5 and 12) had a ring chromosome together with del(6)(q21) as the sole structural anomalies. In none of the tumours could the origin of the ring be identified. Only one head and neck NSCC with a ring chromosome has been reported; that was an r(2) in a complex karyotype (Mark *et al*., 1992). Whether the ring formations play any pathogenetic role or occur as a consequence of the neoplastic process is unknown. Ring chromosomes have been reported in less than 3% of karyotypically abnormal neoplasms (Mitelman, 1994). Among solid tumours, supernumerary rings have been associated with borderline or low malignant mesenchymal tumors, e.g. atypical lipomas or highly differentiated liposarcomas (Heim *et al*., 1988; Mandahl *et al*., 1988*a*), myxoid malignant fibrous histiocytomas (Mandahl *et al*., 1988*b*, 1989), dermatofibrosarcoma protuberans (Bridge *et al*., 1990; Mandahl *et al*., 1990; Örndal *et al*., 1992) and parosteal osterosarcomas (Mertens *et al*., 1993).

Although some aberrations appear to be common to several NSCC types, others are characteristic for particular NSCC subsets. One common denominator is deletion of 6q, often as the sole aberration, which has been detected in 30% of the tumours and in all subtypes except carcinomas in pleomorphic adenoma (Table II). The more specific aberrations are t(6;9)(q21-24;p13-23), detected so far exclusively in adenoid cystic carcinomas, and translocations involving 8q12-13, which seem to be strongly associated with carcinomas in pleomorphic adenoma. The available cytogenic data on head and neck NSCC also indicate that the karyotypic profile of these tumours differs from that of squamous cell carcinomas (SCCs), the predominant tumour type in this region. A subset of SCCs have had pseudo- or near-diploid karyotypes with seemingly random balanced structural rearrangements and/or numerical changes; several arguments have been put forward to interpret these findings with caution, as they may represent stroma cell mutations (see, for example, Jin *et al*., 1993). Other SCCs have, however, had highly complex karyotypes with chromosome numbers in the triploid range. Recurrent aberrations among these tumours include isochromosome 8q, rearrangements of 11q13 (often as a homogeneously staining region), Robertsonian translocations that frequently involve chromosome 15 and loss of chromosome material from chromosome arms 3p, 7q, 8p, 11q and 17p as well as the short arms of the acrocentric chromosomes (Jin *et al*., 1993).

This work was supported by grants from the Swedish Cancer Society, the Swedish Work Environment Fund and the Medical Faculty of Lund University.

**References**

BRIDGE, J.A., NEFF, J.R. & SANDBERG, A.A. (1990). Cytogenetic analysis of dermatofibrosarcoma protuberans. *Cancer Genet. Cytogenet*., **49**, 199-202.

BULLERDIEK, J., VOLLRATH, M., WITTEKIND, C., CASELITZ, J. & BARTNITZKE, S. (1990). Mucoepidermoid tumor of the parotid gland showing a translocation (3;8)(p21;q12) and a deletion (5)(q22) as sole chromosome abnormalities. *Cancer Genet. Cytogenet*., **50**, 161-164.

BULLERDIEK, J., WOBST, G., MEYER-BOLTE, K., CHILLA, R., HAUBRICH, J., THODE, B. & BARTNITZKE, S. (1993). Cytogenetic subtyping of 220 salivary gland pleomorphic adenomas: correlation to occurrence, histological subtype, and in vitro cellular behavior. *Cancer Genet. Cytogenet*., **65**, 27-31.

HEIM, S., MANDAHL, N., RYDHOLM, A., WILLÉN, H. & MITELMAN, F. (1988). Different karyotypic features characterize different clinicopathologic subgroups of benign lipogenic tumors. *Int. J. Cancer*, **42**, 863-867.

HIGASHI, K., JIN, Y., HEIM, S., MANDAHL, N., BIÖRKLUND, A., WENNERBERG, J., DICTOR, M. & MITELMAN, F. (1991*a*). Chromosome abnormalities in a carcinoma in pleomorphic adenoma of the lacrimal gland. *Cancer Genet. Cytogenet*., **55**, 125-128.

HIGASHI, K., JIN, Y., JOHANSSON, M., HEIM, S., MANDAHL, N., BIÖRKLUND, A., WENNERBERG, J., HAMBRAEUS, G., JOHANSSON, L. & MITELMAN, F. (1991*b*). Rearrangement of 9p13 as the primary chromosomal aberration in adenoid cystic carcinoma of the respiratory tract. *Genes Chrom. Cancer*, **3**, 21-23.

ISCN (1991). *Guidelines for Cancer Cytogenetics, Supplement to An International System for Human Cytogenetic Nomenclature*, Mitelman, F. (ed.) Karger: Basle.

JIN, Y. & MERTENS, F. (1993). Chromosome abnormalities in oral squamous cell carcinomas. *Oral Oncol., Eur. J. Cancer*, **29B**, 257-263.

JIN, Y., MANDAHL, N., HEIM, S., BIÖRKLUND, A., WENNERBERG, J. & MITELMAN, F. (1988). Unique karyotypic abnormalities in a squamous cell carcinoma of the larynx. *Cancer Genet. Cytogenet*., **30**, 177-179.

JIN, Y., MERTENS, F., MANDAHL, N., HEIM, S., OLEGÅRD, C., WENNERBERG, J., BIÖRKLUND, A. & MITELMAN, F. (1993). Chromosome abnormalities in eighty-three head and neck squamous cell carcinomas: influence of culture conditions on karyotypic pattern. *Cancer Res*., **53**, 2140-2146.

JOHANSSON, B., HEIM, S., MANDAHL, N., MERTENS, F. & MITELMAN, F. (1993). Trisomy 7 in nonneoplastic cells. *Genes Chrom. Cancer*, **6**, 199-205.

MANDAHL, N., HEIM, S., ARHEDEN, K., RYDHOLM, A., WILLÉN, H. & MITELMAN, F. (1988*a*). Three major cytogenetic subgroups can be identified among chromosomally abnormal solitary lipomas. *Hum. Genet*., **79**, 203-208.

MANDAHL, N., HEIM, S., ARHEDEN, K., RYDHOLM, A., WILLÉN, H. & MITELMAN, F. (1988*b*). Rings, dicentrics, and telomeric association in histiocytomas. *Cancer Genet. Cytogenet*., **30**, 23-33.

MANDAHL, N., HEIM, S., WILLÉN, H., RYDHOLM, A., ENEROTH, M., NILBERT, M., KREICBERGS, A. & MITELMAN, F. (1989). Characteristic karyotypic anomalies identify subtypes of malignant fibrous histiocytoma. *Genes Chrom. Cancer*, **1**, 9-14.

MANDAHL, N., HEIM, S., WILLÉN, H., RYDHOLM, A. & MITELMAN, F. (1990). Supernumerary ring chromosome as the sole cytogenetic abnormality in a dermatofibrosarcoma protuberans. *Cancer Genet. Cytogenet*., **49**, 273-275.

MANDAHL, N., HÖGLUND, M., MERTENS, F., RYDHOLM, A., WILLÉN, H., BROSJÖ, O. & MITELMAN, F. (1993). Cytogenetic aberrations in 188 benign and borderline adipose tissue tumors. *Genes Chrom. Cancer*, **9**, 207-215.

MARK, J., EKEDAHL, C. & DAHLENFORS, R. (1981). Polyclonal chromosomal evolution in a cultured human acinic cell tumour. *Anticancer Res*., **1**, 45-48.

MARK, J., WEDELL, B., DAHLENFORS, R. & STENMAN, G. (1991). Karyotypic variability and evolutionary characteristics of a polymorphous low grade adenocarcinoma in the parotid gland. *Cancer Genet. Cytogenet*., **55**, 19-29.

MARK, J., DAHLENFORS, R., STENMAN, G., BENDE, M. & MELÉN, I. (1992). Cytogenetical observations in two cases of polymorphous low-grade adenocarcinoma of the salivary glands. *Anticancer Res.*, **12**, 1195-1198.

MERTENS, F., HEIM, S., MANDAHL, N., JOHANSSON, B., MERTENS, O., PERSSON, B., SALEMARK, L., WENNERBERG, J., JONSSON, N. & MITELMAN, F. (1991). Cytogenetic analysis of 33 basal cell carcinomas. *Cancer Res.*, **51**, 954-957.

MERTENS, F., MANDAHL, N., ÖRNDAL, C., BALDETORP, B., BAUER, H.C.F., RYDHOLM, A., WIEBE, T., WILLÉN, H., ÅKERMAN, M., HEIM, S. & MITELMAN, F. (1993). Cytogenetic findings in 33 osteosarcomas. *Int. J. Cancer*, **55**, 44-50.

MITELMAN, F. (1994). *Catalog of Chromosome Aberrations in Cancer*, 5th edn, Wiley-Liss: New York.

NORDKVIST, A., EDSTRÖM, S., MARK, J. & STENMAN, G. (1992). Multiple unrelated chromosome abnormalities in a metastatic mucoepidermoid carcinoma of the parotid gland. *Cancer Genet. Cytogenet.*, **61**, 158-161.

ÖRNDAL, C., MANDAHL, N., RYDHOLM, A., WILLÉN, H., BROSJÖ, O., HEIM, S. & MITELMAN, F. (1992). Supernumerary ring chromosomes in five bone and soft tissue tumors of low or borderline malignancy. *Cancer Genet. Cytogenet.*, **60**, 170-175.

SANDROS, J., MARK, J., HAPPONEN, R.-P. & STENMAN, G. (1988). Specificity of 6q− markers and other recurrent deviations in human malignant salivary gland tumors. *Anticancer Res.*, **8**, 637-644.

SREEKANTAIAH, C., KARAKOUSIS, C.P., LEONG, S.P.L. & SANDBERG, A.A. (1992). Cytogenetic findings in liposarcoma correlate with histopathologic subtypes. *Cancer*, **69**, 2484-2495.

STENMAN, G. & MARK, J. (1983). Loss of the Y chromosome in a cultured human salivary-gland adenocarcinoma. *J. Oral Pathol.*, **12**, 458-464.

STENMAN, G., DAHLENFORS, R., MARK, J. & SANDBERG, N. (1982). Adenoid cystic carcinoma: a third type of human salivary gland neoplasm characterized cytogenetically by reciprocal translocations. *Anticancer Res.*, **2**, 11-16.

STENMAN, G., SANDROS, J., DAHLENFORS, R., JUBERG-ODE, M. & MARK, J. (1986). 6q− and loss of the Y chromosome - two common deviations in malignant human salivary gland tumors. *Cancer Genet. Cytogenet.*, **22**, 283-293.

STENMAN, G., SANDROS, J., MARK, J. & EDSTRÖM, S. (1989). Partial 6q deletion in a human salivary gland adenocarcinoma. *Cancer Genet. Cytogenet.*, **39**, 153-156.

UICC (1987). TNM *Classification of Malignant Tumours*. Springer: New York.